# Students’ self-perception of empathy in caring

**DOI:** 10.4102/curationis.v46i1.2364

**Published:** 2023-06-23

**Authors:** Lerato Matshaka

**Affiliations:** 1 Department of Nursing Sciences, Faculty of Health Sciences, University of Johannesburg, Johannesburg, South Africa

**Keywords:** caring, empathy, perceptions, student nurses, mindfulness, awareness

## Abstract

**Background:**

The attribute of empathy leads to more desired patient outcomes. A patient who experiences empathy from student nurses will feel important and cared for. It is vital to know how student nurses perceive themselves in terms of empathy in caring. Thus, self-reflection is a requirement on the part of student nurses in a caring relationship.

**Objectives:**

This study aimed to determine student nurses’ self-perceptions of empathy in caring and compare the third- and fourth-year student nurses’ self-perceptions of empathy in caring.

**Method:**

A quantitative, descriptive and comparative approach was employed in the study. The population was undergraduate student nurses in their third- and fourth-year level of study (*n* = 77), while 56 respondents participated in the study. Ethical approval was obtained prior to commencing with the study. Data were collected by way of the Consultation and Relational Empathy measure questionnaire that consisted of 10 items responded to by using the 5-point Likert scale. Data were analysed by means of descriptive statistics, inferential statistics and *t*-tests.

**Results:**

All the student nurses perceived themselves to have empathy in caring. There was no significant difference in perceptions of empathy in caring by the nurses in their third- and fourth-year level of study.

**Conclusion:**

The results of the study provide insights for nursing education and training to shape and mould the empathy perceived by the student nurses. Future research could focus on the patients’ perspective coupled with the student nurses’ perspective to prevent bias.

**Contribution:**

This paper contributes by adding self perceptions of empathy by student nurses to support best practice in nursing.

## Introduction

Self-perception is an attribute that expresses how the student nurses view themselves in terms of possessing empathy. Self-perception is subjective but it is important because it enhances awareness in the caring encounter with a patient, and awareness enhances empathy (Matshaka, Downing & Poggenpoel 2020:375). Self-perception enhances awareness because it requires student nurses to reflect and to self-introspect, which in turn leads to improvement. A student who has self-awareness is authentic in caring and builds therapeutic relationships with the patients and their families (Matshaka 2021:2; Ndawo 2022:6). Empathy is an effective skill for impactful nurse and patient relationship with notable effects in patient satisfaction and positive patient health outcomes (Jani, Blane & Mercer 2012:252–257; Kim 2018:229–236). Blanco et al. (2020:1–9) reported that patients who are treated with empathy by student nurses report better health outcomes and less hospital stays, than patients who are not treated with empathy. However, media reports still note that caring provided in hospitals is void of empathy (Sowetan Live 2016; The Star 2020). Patients are treated as statistics and diseases where nurses hardly take time to spend with patients and to really provide care with empathy (Bas-Sarmientoa et al. 2019:40–51; Borkar 2014:428–438). It seems as if student nurses, who are the future of nursing, have little to no training on empathy. No follow-ups are made to ensure the transfer of empathy in the clinical area through teaching moments on empathy in the clinical arena (Levett-Jones, Cant & Lapkin 2019:80–94).

Identifying with the patient’s suffering and showing understanding thereof reflects empathy in caring (Ferri et al. 2019:457; Treviza et al. 2015:367–376). The four components of empathy include emotive, moral, cognitive and behavioural affect (Cañas-Lerma et al. 2021:1–9; Mercer & Reynolds 2002:S10). These four components are necessary in producing a competent student nurse who will relate to the patient’s needs. As a result, the student nurse will have competence to identify with the patients. By showing great understanding of the patient’s perspective, the student nurse will show competence to have a cognitive, moral and emotional response (Ferri et al. 2019:367–376; Wang, Cao & Du 2022:1–9).

Morse et al. (1992:75–87) and Gerace (2020:1–2) argue that through the emotive aspect of empathy, the student nurse subjectively experiences the psychological state of the patient. The moral aspect refers to the student nurse who has this internal humane force that motivates them to practise empathy (Fatter & Hayes 2013:502–513; Gerace 2020:1–2; Morse et al. 1992:75–87). Through the cognitive aspect the student nurse understands the patient’s position and experience and can view this objectively (Borkar 2014:428–438; Ferri et al. 2019:457–467; Morse et al. 1992:75–87; Ter Beest, Van Bemmel & Adriaansen 2018:1390–1397). With reference to the behavioural aspect of empathy, the student nurse responds to the need that has been identified to display understanding of the need (Bauchat, Seropian & Jeffries 2016:356–359; Englander & Folkesson 2014:294–313; Morse et al. 1992:75–87). Empathy can also be defined as the ability for student nurses to experience what the patient is experiencing (Gerace 2020:1–2).

Empathy may empower the student nurses to have self-awareness, which enables them to pay attention to the feelings of the patient and this is crucial in caring (Wang et al. 2022:1–9). A student nurse who is aware of the patient’s negative emotional state is empowered to provide relevant care to the patient with the aim of improving the state in which the patient is in (Strekalova et al. 2017:61–79). While empathising with the patient, the student nurse has the ability to separate their emotions from those of the patient. Lamothe et al. (2015:19–28) assert that student nurses can accept these emotions if they have mastered mindfulness.

Through empathy the student nurse shows interest to the patient while communicating with them, they listen for understanding and not just to respond and they display warmth (Mercer & Reynolds 2002:S9–S13). According to Walker and Mann (2016:188–190), she puts on the patient’s shoes to understand and meet their needs. In a therapeutic encounter with the patient, the student nurses aim to understand the patient’s problem from their worldview and to support them and lessen their problems (Mercer & Reynolds 2002:S9–S13).

By implementing empathy in caring the student nurse promotes improved health outcomes, affordable treatment and enhances patient satisfaction (Bauchat et al. 2016:356–359). In addition, empathy encourages patients to adhere to treatment (Flickinger et al. 2015:220–226). Patients show adherence to treatment because they feel valued and understood. Complying with treatment becomes easy because the patients feel acknowledged and that their viewpoint is not ignored (Borkar 2014:428–438). Valuing and respecting the patient through empathy builds trust between the student nurse and the patient. Therefore, should the need arise to inform the patient of any changes in their illness the process becomes easier. Patients who experience empathy in caring participate actively in their healing process and they are more accepting (Kim 2018:229–236).

Situations such as the recent COVID-19 pandemic warranted the nurses and student nurses to show empathy to patients. The global health was under attack by the coronavirus pandemic that claimed lives, and the longstanding devastating impacts of this pandemic will long be remembered (Robbins et al. 2020:2). Hospitals were overburdened (Mbunge 2020:1809–1814) with infected patients who were isolated from families and friends and who needed empathy more than ever before. Thus, student nurses played a crucial role in caring and in ensuring that patients are treated with empathy (Cañas-Lerma et al. 2021:1–9; Taylora, Thomas-Gregory & Hofmeyer 2020:1–4). Empathy in medical students has been explored by Archer and Turner (2019:1–5) and Levett-Jones et al. (2019:80–94) who studied the effectiveness of empathy education for undergraduate student nurses. However, student nurses’ self-perceptions of empathy in caring are yet to be explored. Thus, the aim of this study was to determine student nurses’ self-perceptions of empathy in caring and to compare the self-perceptions among third- and fourth-year level nursing students. Awareness of how student nurses perceive their empathy in caring could provide insights for nurse educators and policymakers regarding ways to overcome the negative reputation and to improve the training of nurses. If student nurses can reflect on their actions, it can lead to corrective measures. In so doing, they contribute to patient satisfaction, patient and nurse relationship and health outcomes.

## Research design and methods

### Study design

A quantitative, descriptive and comparative approach (Gray, Grove & Sutherland 2017:198–208) was employed to identify student nurses’ self-perceptions of empathy in caring and to compare these self-perceptions among third- and fourth-year nursing students.

### Setting

The study was conducted at a higher education institution where 180 first- to fourth-year level students were registered for the undergraduate nursing qualification in Johannesburg, South Africa. The respondents for the study comprised undergraduate student nurses in their third- and fourth-year level of study.

### Study population and sampling strategy

The population of the study was made up of all the third- (*n* = 48) and fourth-year (*n* = 29) student nurses who were purposively selected because they are more exposed to the clinical area and nursing care, and their curriculum is mainly composed of nursing subjects (Matshaka 2018:44). At the first year, the students are introduced to basic nursing and their curriculum is mainly made up of Anatomy, Physiology, Sociology and Psychology (Matshaka 2018:44). In the second year, the student nurses continue with basic nursing and the subjects mentioned here. In their third and fourth years, student nurses focus on nursing subjects and they spend more time in the hospital. Participation was voluntary and 35 of the third-year and 21 fourth-year students completed the survey (Matshaka 2018:44).

### Data collection

To limit bias, data were collected by the researcher who was not involved in the teaching of the third and fourth years. Data were collected by using the existing Consultation and Relational Empathy (CARE) measure questionnaire, which is available for free use provided it is not used for commercial purposes. This questionnaire contained 10 items on a 5-point Likert scale. The response categories of the questionnaire ranged from poor (1) to excellent (5). The Cronbach’s alpha for the CARE measure was 0.97 (Bikker et al. 2015:1–9; Matshaka 2018:90–94). No changes were made to the original instrument.

### Data analysis

The researcher captured the data into an Microsoft Excel spreadsheet and submitted it to the statistician for analysis (Matshaka 2018:53). The statistician used the Statistical Package for Social Science (SPSS) version 23 for descriptive statistics, inferential statistics and *t*-tests (Matshaka 2018:44). Cronbach’s alpha coefficient was calculated to ensure internal consistency (Matshaka 2018:44).

### Ethical considerations

Ethical clearance to conduct this study was obtained from the University of Johannesburg, Faculty of Health Sciences Research Ethics Committee (No. REC-241112-035). Informed consent was obtained in line with ethical requirements of the university.

## Results

The results are presented and discussed based on the three subsections of caring: empathy, compassion and communication. The population was undergraduate student nurses in their third- and fourth-year level of study (*n* = 77), third-year level (*n* = 48), fourth year level (*n* = 29) (Matshaka 2018:44). While 56 respondents participated in the study (Matshaka 2018:44), however, two students did not finish completing the questionnaire, so results from 54 respondents are reported. There were 35 respondents from third-year level and 21 from the fourth-year level (Matshaka 2018:44). Six male (17%) student nurses and 29 female (83%) student nurses participated in the study, with a response rate of 73% in the third-year level of study. One (4.8%) male student nurse and 20 (95%) female student nurses participated in the study in the fourth-year level of study, with a response rate of 72% (Matshaka 2018:44). The minimum age was 19 years and the maximum age was 29 with the mode = 21.00, the median = 22.00 and the mean and standard deviation = 22.17 ± 1.959 (Matshaka 2018:44).

Table 1 provides the 10 questions in the questionnaire together with the responses, means and standard deviation for each question. Table 2 presents the results of the *t*-test.

**TABLE 1:** A summary of the Consultation and Relational Empathy measure results.

How good are you at…?	Construct measured	Good (%)	Very good (%)	Mean ± s.d.
Making the patient feel at ease (introducing yourself, explaining your position, being friendly and warm towards the patient, treating them with respect; not cold or abrupt)	Compassion	22.3	77.8	4.00 ± 0.727
Letting patients tell their story (giving them time to fully describe their condition in their own words; not interrupting rushing or diverting them)	Communication	35.2	64.8	3.80 ± 0.833
Really listening (paying close attention to what the patients were saying, not looking at the notes or computer as they were talking)	Communication	44.5	55.6	3.67 ± 0.824
Being interested in the patient as a whole person (asking/knowing relevant details about their life, their situation; not treating them as ‘just a number’)	Empathy	37.1	63.0	3.74 ± 0.805
Fully understanding patient’s concerns (communicating that you have accurately understood their concerns and anxieties, not overlooking or dismissing anything)	Empathy	48.2	51.8	3.57 ± 0.767
Showing care and compassion (seeming genuinely concerned, connecting with the patient on a human level; not being indifferent or ‘detached’)	Compassion	27.8	72.2	3.94 ± 0.763
Being positive (having a positive approach and a positive attitude; being honest but not negative about the patient’s problem)	Empathy	27.8	72.3	3.80 ± 0.737
Explaining things clearly (fully answering their questions; explaining clearly, giving them adequate information; not being vague)	Communication	51.8	48.2	3.46 ± 0.818
Helping the patient to take control (exploring with them what they can do to improve their health themselves; encouraging rather than lecturing ‘the patient’)	Empathy	46.3	53.7	3.57 ± 0.838
Making a plan of action with the patient (discussing the options, involving them in decisions as much as they want to be involved; not ignoring their views)	Communication	61.1	38.9	3.39 ± 0.834

*Source*: Adapted from Matshaka, L., 2018, ‘Student nurses’ perceptions of the relationship between mindfulness and caring’, Unpublished masters dissertation, pp. 1–182, University of Johannesburg, Johannesburg

**TABLE 2:** Consultation and Relational Empathy measure *t*-tests.

Level of study	*n*	Mean ± s.d.	*S*-error
3rd-year	34	3.7882 ± 0.44228	0.07585
4th-year	20	3.5350 ± 0.68462	0.15309

*Source*: Matshaka, L., 2018, ‘Student nurses’ perceptions of the relationship between mindfulness and caring’, Unpublished masters dissertation, pp. 1–182, University of Johannesburg, Johannesburg

## Discussion

The results of the study indicate that student nurses perceive themselves to have empathy in caring. The highest mean for the Compassion section was 4.00, with the standard deviation of 0.727 (Matshaka 2018:90–94). This is in contrast to the results of the study conducted by Kim (2018:229–236) who found a lower mean of 3.44. The highest percentage (53.7%) for this section lies in the ‘very good’ category. Compassion is an important aspect that brings action after noting the need with which the patient presents. It brings about the behavioural aspect of empathy (Morse et al. 1992:75–87). The mean for empathy was greater than 2.5, indicating that the student nurses believe they possess empathy (Gray et al. 2017:538). They view themselves as able to place themselves in the patient’s shoes to understand the patient’s suffering.

The means for Empathy, Compassion and Communication were relatively high; thus, it can be assumed that these questions represented the constructs that were being measured (Gray et al. 2017:538). The standard deviation for this question of the compassion construct is relatively low, which also shows low deviation from the mean (Polit & Beck 2017:361–362).

The results of the Levene’s test recorded a significant value of 0.035. As the significance level of Levene’s test is less than 0.05, the researcher used the *t*-value (1.482) in the second line, which states that equal variances are not assumed (Pallant 2016:208). The significance (two-tailed) value is 0.149, which is greater than 0.05. This suggests that there is no significant difference between the third- and fourth-year levels of study in terms of caring. There was no significant difference in the mean scores of the third and fourth years in terms of empathy (refer to Table 2). The third year showed slightly higher mean scores on the CARE measure (3.7882 ± 0.44228) than the fourth year (3.5350 ± 0.68462) with *t* (52) = 1.482 and *p* = 0.104. The mean difference was 0.25324. The 95% confidence interval of the difference was recorded as follows: Low = –0.05410 and Upper = 0.60293.

There were no significant differences in perceptions of empathy in caring among the third- and fourth-year level student nurses. This is not consistent with previous studies that show that final year students tend to display more empathy (Magalhães et al. 2011:52–59; Ulloque et al. 2019:81–86). On the contrary, Ferri et al. (2017:22–30) argue that the empathy level of students decline as they progress with their levels of study. There were also no significant differences in perceptions of empathy among males and females. These results are inconsistent with previous studies that showed that females tend to portray more empathy than males (Chen et al. 2012:305–311; Ferri et al. 2017:22–30; Hojat & Gonnella 2015:344–350; Jia-Ru, Yan-Xuea & Wen-Nv 2022:1–9; O’Sullivan et al. 2017:[online]); however, the results were similar to other results (Deng et al. 2023:1–7; Li et al. 2018:241–250; Paro et al. 2012:73–80).

### Strengths and limitations

The study focused on a one-sided self-report of empathy of student nurses. Future research could focus on both angles being the student nurses’ self-report and the patients’ rating on the empathy provided by student nurses. Further research could also include mixed methods as means of establishing student nurses’ self-perceptions of empathy.

### Recommendations

Empathy is a crucial attribute to ensure that the student nurse, the patient, the hospital, and the health system have better outcomes. Student nurses must be taught to intentionally empathise with patients by practically understanding the world of the patients through co-presence, while setting their assumptions aside. Simulation laboratories can be used to cultivate empathy in student nurses by using gaming and virtual and artificial reality (Pandey, Mohan & Chowdhury 2022:119–125). More research is needed and could focus on measuring empathy from student nurses at first-year level through to the fourth-year level to monitor empathy development during the nursing qualification. Moreover, future research could incorporate important views of the patients on the empathy received from student nurses. The implications of this research to practice are that education needs to be harnessed when in the clinical area by nursing professionals, clinical preceptors and mentors by using teachable moments to cultivate empathy in student nurses and the patients. This can be performed practically with patients.

In summary, the study recommends the following:

- Empathy should be taught, measured and assessed in a simulation laboratory setting.
- Platforms such as wiki, blogs can be used where student nurses can write reflective journals of their experiences of empathy.
- Mentors and clinical preceptors can be used in a clinical setting to focus on teaching empathy through teaching moments by the patient’s bed side and not only focus on clinical skills.

## Conclusion

The aim of the study was to determine the perceptions of empathy in caring by student nurses and to compare the perceptions among the third- and fourth-year level of student nurses. Student nurses perceive themselves to have empathy in caring and results showed high means and low standard deviations. There was no significant difference in the perceptions of the third and fourth years. These results are noteworthy because the student nurses in third- and fourth-year level of study have both been exposed to the clinical area. Both groups had spent time with the patients, and these results show that they have reflected on the interactions they have had with these patients, and they consider that they have empathy. The empathy that student nurses perceive themselves to have, can be used as good ground to cultivate empathy that can be realisable even to the patients and other members of the multi-disciplinary team. Nurse educators could be more deliberate in teaching empathy to the student nurses in more practical manners. Further research could focus on measuring empathy even from the patients’ and other members of the multi-disciplinary team’s perspective, to determine their perceptions of the empathy provided by student nurses in caring.
